# Analysis of the effects of azithromycin combined with budesonide and children’s Chaiqiao Qingre granules on the efficacy and improvement of clinical symptoms of mycoplasma pneumonia in children

**DOI:** 10.1097/MD.0000000000040557

**Published:** 2025-06-20

**Authors:** Gaowa Bao

**Affiliations:** a Department of Pediatrics, Affiliated Hospital of Inner Mongolia University for Nationalities Tongliao, Inner Mongolia, China.

**Keywords:** azithromycin, budesonide, clinical symptoms, pediatric mycoplasma pneumonia, Xiao’er Chiqiao Qingre Granules

## Abstract

To explore the effects of azithromycin combined with budesonide and children’s Chaiqiao Qingre granules on the efficacy and improvement of clinical symptoms of mycoplasma pneumonia in children. A retrospective analysis was conducted on the case data of 115 pediatric patients diagnosed with mycoplasma pneumonia admitted to our pediatric department between February 2023 and February 2024. Based on their treatment regimen, the patients were categorized into an observation group (OG) (treated with azithromycin in combination with budesonide and Xiao’er Chiqiao Qingre Granules, totaling 60 cases) and a control group (CG) (treated with azithromycin combined with budesonide, 55 cases). A comparative analysis was performed between the 2 groups regarding the total treatment effectiveness, duration until relief of clinical symptoms, alterations in immunoglobulin levels, pulmonary function indicators, serum C-reactive protein (CRP), serum procalcitonin (PCT) levels before and after treatment, and the incidence of adverse reactions. The total treatment effectiveness of OG markedly surpassed that of the CG (96.67% vs 80.00%) (*P* < .05), and the time for relief of various clinical symptoms in the OG (fever, cough, and pulmonary moist rales) was notably shorter (*P* < .05). After treatment, the levels of immunoglobulins, pulmonary function indicators, serum CRP, and serum PCT in both groups of children showed significant improvement compared to before treatment, but the improvement of OG was more significant compared to the CG (*P* < .05). There was no significant difference in the incidence of adverse reactions between the 2 groups of children (*P* > .05). Azithromycin combined with budesonide and children’s Chaiqiao Qingre granules can effectively improve the clinical symptoms of children with mycoplasma pneumonia, enhance immune function, improve pulmonary function, reduce inflammatory response, and reduce the incidence of adverse reactions, which is worthy of clinical promotion and application.

## 1. Introduction

Pediatric mycoplasma pneumonia stands as a prevalent pediatric ailment, primarily stemming from *Mycoplasma pneumoniae* infection. This organism’s metabolic byproducts trigger extensive inflammatory responses, yielding symptoms like fever, cough, chest pain, and tightness. Untreated, it can ravage the respiratory system and vital organs, amplifying the risk of infant mortality.^[1,2]^ Given children’s vulnerable immunity, the disease progresses swiftly postinfection. Clinical X-rays often reveal lung hilum shadow thickening and prominent pulmonary wet rales. Untimely intervention may precipitate refractory pneumonia, imperiling young lives.^[3]^ Swift clearance of mycoplasma infection upon detection is thus imperative, averting recurrent bouts and safeguarding children’s physical well-being and developmental trajectory.^[4]^

With the emergence of second-generation macrolide antibiotics, azithromycin has emerged as a cornerstone in the treatment of pediatric mycoplasma pneumonia. Its ability to impede bacterial protein synthesis renders it bacteriostatic. Complementing azithromycin, clinical practice often integrates nebulized budesonide inhalation, enhancing efficacy and expediting recovery in afflicted children.^[5,6]^ Nonetheless, sole reliance on Western medicine frequently yields suboptimal outcomes due to its prolonged half-life, often triggering various adverse reactions in pediatric patients.^[7]^ Consequently, the adoption of traditional Chinese medicine or Chinese patent medicine is gaining traction, particularly in cases where antibacterial agents alone prove ineffective.^[8]^ Traditional Chinese medicine posits mycoplasma pneumonia within the realm of “pneumonia cough asthma,” attributing it to external pathogen invasion of lung tissue. Xiao’er Chiqiao Qingre Granules, primarily comprising light soybean, peppermint, scutellaria, and pinellia, exhibit notable efficacy in lung clearance, phlegm resolution, cough suppression, and asthma relief–aligning with mycoplasma pneumonia treatment principles.^[9,10]^ However, scant clinical documentation exists on the combined use of azithromycin, budesonide, and Xiao’er Chiqiao Qingre Granules in pediatric mycoplasma pneumonia. This study scrutinizes the therapeutic impact of combining azithromycin and budesonide with Xiao’er Chiqiao Qingre Granules in pediatric mycoplasma pneumonia, endeavoring to enrich treatment options for this pediatric condition.

## 2. Materials and methods

### 2.1. Clinical data

This study was approved by the Ethics Committee of Affiliated Hospital of Inner Mongolia University for Nationalities. A retrospective analysis of the case data of 115 pediatric mycoplasma pneumonia patients admitted to our pediatric department from February 2023 to February 2024 was conducted. According to the treatment plan of the patients, they were divided into the observation group (OG) (treated with the combination of azithromycin and budesonide with Xiao’er Chiqiao Qingre Granules) with 60 cases and the control group (CG) (treated with azithromycin and budesonide) with 55 cases. Inclusion criteria: Children diagnosed with mycoplasma pneumonia^[11]^; children under 12 years old; children with complete medical records. Exclusion criteria: Children with concomitant viral or bacterial infections; children with severe liver or kidney dysfunction; children with malignant tumor diseases; children with immunodeficiency diseases. This experiment has been approved by the hospital ethics committee and complies with the Helsinki Declaration.

### 2.2. Treatment methods

Both groups of children were treated with intravenous infusion and oral administration of azithromycin and nebulized inhalation of budesonide. Azithromycin (Pfizer Pharmaceuticals Limited, National Drug Approval Number: J20140073) was used at a dose of 10 mg/kg, intravenous infusion for 3 days, then stopped for 4 days, and switched to oral azithromycin, once daily for 3 days, and stopped for 4 days. Budesonide (Hubei Gedian Renfu Pharmaceutical Co. Ltd, National Drug Approval Number: H20103795) was administered by nebulized inhalation, 1 mg per time, twice a day, for 7 days. In the OG, Xiao’er Chiqiao Qingre Granules (Jichuan Pharmaceutical Group Co. Ltd, National Drug Approval Number: Z20050154) were added on the basis of the CG for treatment. It was taken orally with warm water after meals, 3 times a day, according to the corresponding age and dosage in the instructions, for 7 days.

### 2.3. Observation indicators

#### 2.3.1. Observation index

Comparison of the total effective rate of treatment in the 2 groups of patients: Children who have recovered with normal body temperature, disappearance of clinical symptoms, normal laboratory examination indicators, disappearance of pulmonary wet rales, and disappearance of chest X-ray images are considered cured; children with basically normal body temperature, significant improvement in clinical symptoms, laboratory examination indicators approaching normal, disappearance or significant reduction of pulmonary wet rales, and significant shrinkage of chest X-ray images are considered markedly effective; children with decreased body temperature but still have fever, with some relief in the above clinical examination symptoms are considered effective; children whose condition has not improved or worsened are considered ineffective. The total effective rate = (cured + markedly effective + effective)/ total number of cases × 100%; Time to relieve clinical symptoms. The duration of fever, cough, and pulmonary rales were compared between the 2 groups; Immunoglobulin levels. Three milliliters of venous blood were collected from the elbow of the patient before treatment and 1 week after treatment, and immunoturbidimetry was used to determine the levels of immunoglobulin A (IgA), immunoglobulin G (IgG), and immunoglobulin M (IgM) (reagent kit purchased from Ningbo Prui Biotechnology Co. Ltd); pulmonary function indicators: pulmonary function tests were performed before and after treatment to detect changes in forced expiratory volume in 1 second (FEV_1_), forced vital capacity (FVC), and peak expiratory flow (PEF) in children; Serum inflammatory indicators: Venous blood was collected from the elbow vein of the patient, placed in a vacuum collection tube, mixed evenly, centrifuged using a centrifuge, the supernatant was taken, and sent for inspection. C-reactive protein (CRP) was detected using immunoturbidimetry, and procalcitonin (PCT) was detected using a double-antibody sandwich enzyme-linked immunosorbent assay; occurrence of adverse reactions. The occurrence of adverse reactions such as gastrointestinal reactions, rash, headache, and tachycardia in the 2 groups of patients during the treatment period was recorded. The total incidence of adverse reactions = (gastrointestinal reactions + rash + headache + tachycardia)/ total number of cases in this group × 100%.

### 2.4. Statistical analysis

Data were analyzed using SPSS 22.0 software. Measurement data that followed a normal distribution were presented as mean ± standard deviation ($\bar{x}\pm s$), and intergroup comparisons were analyzed using the *t*-test. Count data were presented as percentages (%) and compared between groups using the χ² test. *P* < .05 was considered statistically significant.

## 3. Results

### 3.1. Comparison of general information

The 2 groups were comparable due to insignificant difference in gender, age, body weight, course of disease, or clinical symptoms (*P* > .05, Table 1).

**Table 1:** Comparison of general information.

Variable	Observation group (n = 60)	Control group (n = 55)	*t*/χ²	*P*
Gender				
Male	33 (55.00)	31 (54.39)	0.004	.947
Female	27 (45.00)	26 (45.61)		
Age (yr)				
≤7	40 (66.67)	35 (63.64)	0.016	.733
>7	20 (33.33)	20 (36.36)		
Weight (kg)				
≤20	37 (61.67)	31 (56.36)	0.334	.563
>20	23 (38.33)	24 (43.64)		
Course of disease (d)				
≤3	24 (40.00)	24 (43.64)	0.156	.693
>3	36 (60.00)	31 (56.36)		
Severity of disease				
Mild	28 (46.67)	27 (49.09)	0.119	.943
Moderate	22 (36.67)	20 (36.36)		
Severe	10 (16.66)	8 (14.55)		

### 3.2. Comparison of total effective rate of treatment

The total effective rate of treatment in the OG was significantly higher than that in the CG (96.7% vs 83.6%) (*P* < .05, Table 2).

**Table 2:** Comparison of total effective rate.

Therapeutic effect	Observation group (n = 60)	Control group (n = 55)	χ²	*P*
Cured	38 (63.33)	25 (45.45)	−	−
Significantly effective	14 (23.33)	10 (18.18)		
Effective	6 (10.00)	9 (16.36)	−	−
Ineffective	2 (3.33)	11 (20.00)	−	−
Total effective rate	58 (96.67)	44 (80.00)	7.950	.005

### 3.3. Comparison of clinical symptom scores before and after treatment

Before treatment, there was no statistically significant difference in clinical symptom scores between the 2 groups of children (*P* > .05). After treatment, the clinical symptom scores of both groups were significantly lower than those before treatment, and the decline in OG was more significant (*P* < .05, Table 3).

**Table 3:** Comparison of clinical symptom relief time.

Variable	Observation group (n = 60)	Control group (n = 55)	χ²	*P*
Fever (d)	3.18 ± 0.56	4.91 ± 0.7	8.577	<.001
Cough (d)	5.02 ± 0.61	7.16 ± 0.69	17.65	<.001
Pulmonary moist rales (d)	9.19 ± 0.82	12.08 ± 1.15	15.61	<.001

### 3.4. Comparison of immunoglobulin levels before and after treatment in 2 groups of patients

**Figure 1.**
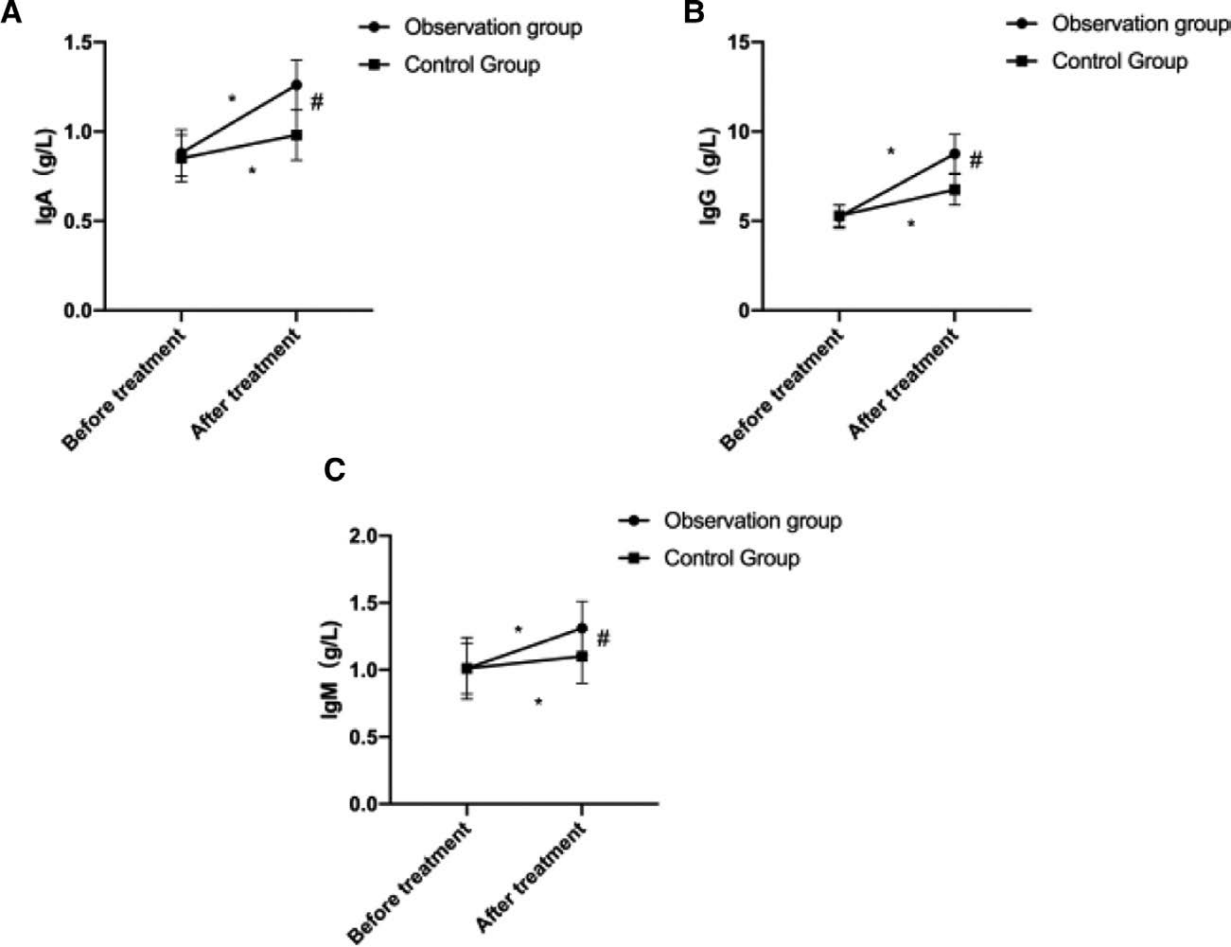
Comparison of immunoglobulin levels before and after treatment (A) Comparison of IgA levels. (B) Comparison of IgG levels. (C) Comparison of IgM levels. * indicates a statistically significant difference within the group before and after treatment (*P* < .05), # indicates a statistically significant difference between groups after treatment (*P* < .05).

Before treatment, no significant difference was detected in serum levels of IgA, IgG, and IgM between the 2 groups (*P* > .05). After 1 week of treatment, all levels were observed elevated in both groups, and those in OG were higher than those in the CG (*P* < .05, Fig. 1).

### 3.5. Comparison of pulmonary function before and after treatment between 2 groups

**Figure 2.**
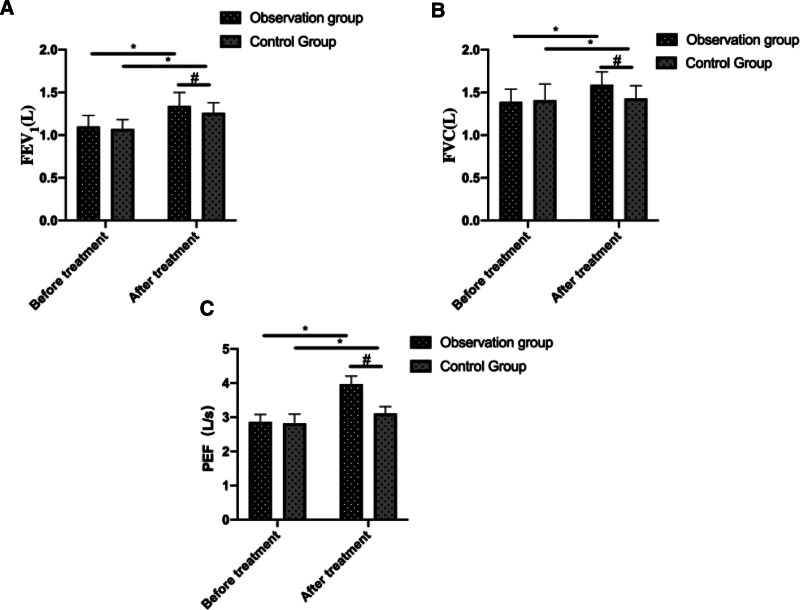
Comparison of pulmonary function before and after treatment in 2 groups. (A) Comparison of FEV_1_ levels before and after treatment in 2 groups of patients. (B) Comparison of FVC levels before and after treatment in 2 groups of patients. (C) Comparison of PEF levels before and after treatment in 2 groups of patients. * indicates a statistically significant difference within the group before and after treatment (*P* < .05), # indicates a statistically significant difference between groups after treatment (*P* < .05).

Before treatment, there was no significant difference in FEV_1_, FVC, and PEF levels between the 2 groups of patients (*P* > .05). After treatment, all levels were significantly improved in both groups, with a more evident improvement presented in OG compared to CG (*P* < .05, Fig. 2).

### 3.6. Comparison of serum inflammatory factors before and after treatment in 2 groups

**Figure 3.**
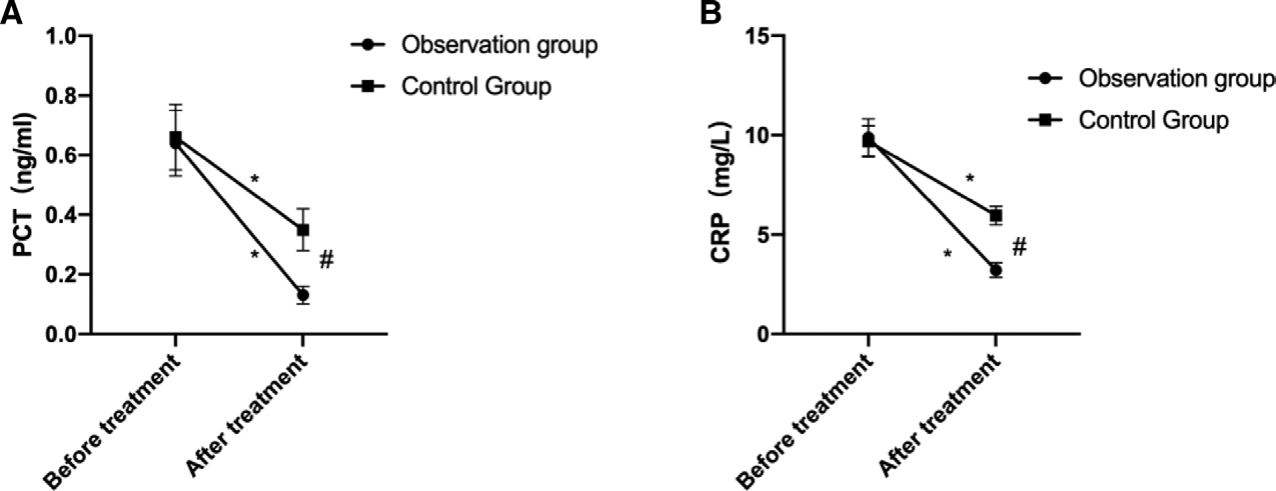
Comparison of serum inflammatory factors before and after treatment between 2 groups. (A) Comparison of PCT levels before and after treatment between 2 groups. (B) Comparison of CRP levels before and after treatment between 2 groups. * indicates a statistically significant difference within the group before and after treatment (*P* < .05), # indicates a statistically significant difference between groups after treatment (*P* < .05).

Before treatment, no major differences are identified in serum PCT and CRP levels between the 2 groups (*P* > .05). After treatment, the levels went significantly lower in both groups (*P* < .05), while such decline was more prominent in the OG than CG (*P* < .05, Fig. 3).

### 3.7. Comparison of adverse reactions during treatment

There was no evident difference regarding incidence of adverse reactions between the 2 groups of children during treatment (*P* > .05), indicating that the combination of azithromycin and budesonide with Xiao’er Chiqiao Qingre Granules did not increase the incidence of adverse reactions in children (*P* < .05, Table 4).

**Table 4:** Comparison of adverse reactions.

Variable	Observation group (n = 60)	Control group (n = 55)	χ²	*P*
Muscle pain	2 (3.33)	1 (1.82)	−	−
Nausea and vomiting	1 (1.67)	1 (1.82)	−	−
Arrhythmia	1 (1.67)	0	−	−
Aspiration pneumonia	0	0	−	−
Overall incidence rate	4 (6.67)	2 (3.64)	0.533	.465

## 4. Discussion

*M pneumoniae* infection is a prevalent respiratory tract disease. This pathogen, lacking a cell wall, can adhere to epithelial cell surfaces via respiratory mucosa, leading to damage to bronchial and pulmonary tissues. Its primary mode of transmission is through droplets.^[11]^ Children and adolescents, in their developmental stage, are particularly vulnerable due to their immature lung development.^[12]^ Symptoms may include persistent cough, fever, and sore throat. Without timely treatment, the infection can escalate to become refractory.^[13]^

Currently, antibiotics are widely employed in the clinical management of pediatric mycoplasma pneumonia to mitigate disease progression. Azithromycin, a second-generation macrolide antibiotic, is the preferred choice for pediatric *M pneumoniae*. It boasts potent antibacterial activity, a broad spectrum, and the ability to inhibit peptidyl transferase transfer reaction, rendering the pathogen inactive.^[14]^ Nevertheless, due to inter-individual variability, this medication may not effectively control inflammation in some children. Budesonide suspension exerts robust local anti-inflammatory effects by competitively inhibiting histamine H receptors. It stabilizes lysosomal membranes, endothelial cells, and smooth muscle cells, inhibits bronchoconstrictive substance synthesis, and suppresses immune responses, thereby reducing the release of allergic mediators. This is beneficial for relaxing smooth muscles and alleviating clinical symptoms in children. Nebulized inhalation therapy offers notable advantages, as the drug can directly target the lesion site, inhibit inflammatory factor release, and modulate immune responses.^[15,16]^ However, given the protracted nature of mycoplasma pneumonia, prolonged Western medicine usage may also precipitate certain side effects. With the widespread adoption of azithromycin in pediatric infectious disease treatment, the issue of drug resistance has gradually surfaced, resulting in diminished clinical efficacy. In instances where sole antibacterial drug usage proves ineffective, the utilization of traditional Chinese medicine or Chinese patent medicine is increasingly prevalent.^[17]^ Traditional Chinese medicine does not classify “mycoplasma pneumonia” as a distinct disease entity. Instead, based on children’s clinical presentations, it is typically categorized under pneumonia and asthma diseases. Traditional Chinese medicine posits that this ailment primarily arises from children’s delicate organs, rendering them susceptible to exogenous pathogens like wind-cold, culminating in symptoms such as cough, fever, and sputum production.^[18]^ Adopting the approach of clearing the lungs and expelling pathogens is advisable. Xiao’er Chiqiao Qingre Granules, a frequently used Chinese patent medicine in pediatrics, have demonstrated efficacy in acute upper respiratory tract infections in children.^[19]^

In our research, we examined the effectiveness of combining azithromycin and budesonide with Xiao’er Chiqiao Qingre Granules in treating pediatric mycoplasma pneumonia. The findings revealed that following treatment, the overall efficacy rate in the OG was significantly higher than that in the CG. Additionally, the duration of cough disappearance, wheezing disappearance, fever reduction, and resolution of pulmonary moist rales were all notably shorter in the OG compared to the CG. These results affirm that the combined therapy of azithromycin and budesonide with Xiao’er Chiqiao Qingre Granules can swiftly alleviate local symptoms associated with mycoplasma pneumonia infection in children, shorten the disease duration, and enhance clinical efficacy. Moreover, our study demonstrated that posttreatment, both groups exhibited significantly reduced levels of PCT and CRP compared to pretreatment levels, with the OG showing notably lower levels of these serum inflammatory markers than the CG. PCT, a nonspecific anti-inflammatory factor, is typically present at extremely low or undetectable levels in the body under normal circumstances. However, during bacterial infection, PCT levels can sharply rise within a brief period. Furthermore, recent research has highlighted the importance of detecting PCT levels in the early diagnosis of mycoplasma pneumonia.^[20]^ CRP, an acute-phase reactant protein, begins to elevate 6 to 8 hours postinfection, peaking 24 to 48 hours thereafter, and swiftly declines upon infection resolution, making it an ideal marker for early mycoplasma pneumonia diagnosis.^[21]^ The findings of this study affirm that the combined administration of azithromycin and budesonide with Xiao’er Chiqiao Qingre Granules effectively suppresses the inflammatory response in children with *M pneumoniae* infection. This effect may stem from the notable anti-inflammatory properties of various active ingredients present in Xiao’er Chiqiao Qingre Granules, including Scutellaria baicalensis, Platycodon grandiflorus, Forsythia suspensa, Fructus, Mentha, and Rheum palmatum. Concurrent usage of the 2 drugs in the CG significantly amplifies the anti-inflammatory response. Furthermore, we conducted a comparative analysis of immunoglobulin levels in both groups of children. The results revealed that posttreatment, serum levels of IgA, IgG, and IgM were elevated in both groups, with higher levels observed in the OG compared to the CG. Immunoglobulins are proteins with immune functions synthesized by immune cells in response to stimulation by pathogenic microorganisms or antigens. Following *M pneumoniae* infection in children, B cells release antibodies and immunoglobulins, initiating a pathological immune response.^[22]^ Among these, IgA serves as a primary immunoglobulin in preventing respiratory tract infections, IgG correlates closely with the progression and infection status of *M pneumoniae* in children, while IgM is among the earliest antibodies detected in humoral immunity. This study suggest that combining Xiao’er Chiqiao Qingre Granules with azithromycin and budesonide enhances immune function in affected children, thereby helping to mitigate the worsening of inflammation. Furthermore, we noted that following treatment, the pulmonary function indicators FVC, PEF, and MMF in the OG were notably higher than those in the CG, indicating that Xiao’er Chiqiao Qingre Granules effectively preserve lung function in affected children. This effect may be attributed to the immunostimulatory and lung-protective properties of various Chinese herbal medicines found in Xiao’er Chiqiao Qingre Granules, including Forsythia suspensa, Platycodon grandiflorus, Bupleurum chinense, Rheum palmatum, Artemisia annua, and Magnolia officinalis. Additionally, there were no severe adverse reactions reported in either group during the treatment period, confirming the safety of the treatment regimen used in this study. This study has several limitations. First, being a retrospective analysis, it inherently carries the potential for selection bias. Additionally, the relatively small sample size may limit the generalizability of the findings. The study was also conducted at a single center, potentially affecting its applicability to broader populations. Finally, the absence of long-term follow-up prevents the assessment of sustained effects of the treatment over time. Future randomized controlled trials with larger sample sizes, multicenter approaches, and extended follow-up periods are recommended to provide more robust evidence for the efficacy and safety of this combination therapy.

In summary, the combined therapy of azithromycin and budesonide with Xiao’er Chiqiao Qingre Granules demonstrates significant clinical efficacy in managing pediatric mycoplasma pneumonia. This approach helps alleviate inflammatory responses, enhances immune function in affected children, expedites the improvement of clinical symptoms, and facilitates the recovery of lung function. Furthermore, its favorable safety profile suggests its potential for wider clinical application.

## Author contributions

**Conceptualization:** Gaowa Bao.

**Data curation:** Gaowa Bao.

**Formal analysis:** Gaowa Bao.

**Investigation:** Gaowa Bao.

**Methodology:** Gaowa Bao.

**Supervision:** Gaowa Bao.

**Validation:** Gaowa Bao.

**Writing – original draft:** Gaowa Bao.

**Writing – review & editing:** Gaowa Bao.
